# Physiological mechanisms associated with the use of a passive heat intervention: positive implications for soccer substitutes

**DOI:** 10.1007/s00421-023-05381-3

**Published:** 2023-12-22

**Authors:** Gavin Cowper, Stuart Goodall, Kirsty M. Hicks, Louise Burnie, Kai T. Fox, Ashleigh Keenan, Enrico De Martino, Marc A. Briggs

**Affiliations:** 1 Faculty of Health and Life Sciences, Northumbria University, Newcastle upon Tyne, NE1 8ST UK; 2 Neuroplasticity and Pain (CNAP), Department of Health Science and Technology, Faculty of Medicine, Aalborg University, Aalborg, Denmark

**Keywords:** Core temperature, Exercise, Muscle temperature, Passive heating, Warm-up

## Abstract

**Purpose:**

Soccer substitutes are exposed to periods of limited activity before entering match-play, likely negating benefits of active warm-ups. This study aimed to determine the effects of using a passive heat intervention following a pre-match, and half-time warm-up, on muscle and core temperature in soccer players during ambient (18 °C) and cold (2 °C) conditions.

**Methods:**

On four occasions, 8 male players, completed a pre-match warm-up, followed by 45 min of rest. Following this, participants completed a half-time re-warm-up followed by an additional 45 min of rest, simulating a full match for an unplaying substitute. During periods of rest, participants wore either standardised tracksuit bottoms (CON), or heated trousers (HEAT), over typical soccer attire.

**Results:**

Vastus lateralis temperature declined less in HEAT compared to CON following the 1st half in 2 °C (Δ − 4.39 ± 0.81 vs. − 6.21 ± 1.32 °C, *P* = 0.002) and 18 °C (Δ − 2.48 ± 0.71 vs. − 3.54 ± 0.88 °C, *P* = 0.003). These findings were also observed in the 2nd half for the 2 °C (Δ − 4.36 ± 1.03 vs. − 6.26 ± 1.04 °C, *P* = 0.002) and 18 °C (Δ − 2.85 ± 0.57 vs. − 4.06 ± 1 °C, *P* = 0.018) conditions. In addition, core temperature declined less in HEAT compared to CON following the 1st (Δ − 0.41 ± 0.25 vs. − 0.84 ± 0.41 °C, *P* = 0.037) and 2nd (Δ − 0.25 ± 0.33 vs. − 0.64 ± 0.34 °C, *P* = 0.028) halves of passive rest in 2 °C, with no differences in the 18 °C condition. Perceptual data confirmed that participants were more comfortable in HEAT vs. CON in 2 °C (*P* < 0.01).

**Conclusions:**

Following active warm-ups, heated trousers attenuate the decline in muscle temperature in ambient and cold environments.

## Introduction

Prior to exercise, a warm-up routine has been suggested to be imperative for task readiness, with the anticipation that it will enhance performance (Bishop 2003). A meta-analysis examining the influence of an active warm-up on subsequent performance showed that ~ 79% of research has demonstrated positive effects on physical performance (Fradkin et al. 2010). The key benefits of an active warm-up are increased muscle temperature (T_muscle_) and core temperature (T_core_) (Bishop 2003). Such a rise in T_muscle_, elicits various physiological benefits, including an increased speed of contraction and relaxation of muscle fibres, increased anaerobic metabolic capacity, nerve conduction enhancements in both the peripheral and central nervous system and improvement in maximal power output (Mohr et al. 2004; Sargeant 1987). However, lengthy periods between an active warm-up and subsequent exercise performance (transition periods), cause a decline in T_muscle_ and T_core_, thereby reducing performance capability (Cowper et al. 2022). As such, methods are needed to maintain the benefits of a warm-up during transition periods.

The application of passive heating garments has been shown to reduce the decline in T_muscle_ and T_core_ during lengthy transition periods, enhancing specific sporting performances (Cowper et al. 2021, 2022; Faulkner et al. 2013a, b). However, only limited studies have examined the effects of longer duration performance (> 5 min) using passive heating devices, which might be due to previous work using hot showers/baths have found a detrimental effect on long duration performance, at ambient temperatures (Gregson et al. 2002). During long duration activity, the detrimental effects caused by passive heating during the transition period have been attributed to a lower heat-storage capacity and earlier attainment of critical core temperature (Fortney et al. 1984; Nadel 1987). However, in colder environments, core temperature would be more likely to decrease, thus increasing the time to reach a critical core temperature during subsequent exercise performance. Thus, in such scenarios, passive heat interventions would be beneficial in attenuating the drop in bodily temperatures, with the potential of a positive impact on performance (Marino 2002).

Often, substitute players in a range of competitive sports, experience a lengthy duration of limited activity before match-play. In relation to soccer specifically, it is common for players to experience up to 45 min of passive rest between the pre-match warm-up and the half-time re-warm-up, prior to potentially entering competitive play. During this period of passive rest, a decline in T_muscle_ and T_core_ might be experienced, which are likely to be greater in colder environmental conditions. Cold environments (~ 2 °C) are frequently encountered during evening kick-off times throughout winter months in the United Kingdom. Thus, methods are needed to support substitutes in maintaining T_muscle_ and *T*_core_ during such long transition periods. It is common for soccer substitutes to perform brief warm-ups, at a submaximal intensity, throughout a match. However, combined with the long periods of passive sitting, these are unlikely to prevent declines in temperature and players are unlikely to be able to capitalise on the benefits of any pre-game warm-up. Furthermore, practitioners need to reduce the possibility of additional fatigue by carrying out multiple re-warming exercises throughout a transition period. Thus, there is an opportunity for practical methods of passive heating to be used to defend against the likely decline in muscle and core temperature, with the ambition of an improved subsequent performance. Such benefits may be applicable to other team sports, where substitutes experience lengthy passive rest periods post warm-up and in particular, sports which are frequently performed in cold environments.

Accordingly, the aim of this study was to (i) compare T_muscle_ and T_core_ between the first and second half of a simulated soccer match for unused substitutes, and (ii) examine these temperature differences following the use of heated trousers, in two different environmental conditions.

## Methods

### Participants

Eight male soccer players (mean ± SD, age 28.8 ± 7.0 years, stature 182.0 ± 4.1 cm, body mass 77.6 ± 4.7 kg, vastus lateralis (VL) depth 2.68 ± 0.2 cm, VL subcutaneous fat depth 0.59 ± 0.1 cm) volunteered to participate in this study. The population training status was defined as a player who regularly completes in a high level of competitive soccer (semi-professional). A sample size of eight was calculated using a change in mean T_muscle_ of the VL, a crossover design in a similar population and the SD of T_muscle_ between HEAT and CON (± 0.4 °C). A statistical power of 0.8 and the smallest worthwhile improvement in T_muscle_ of 1% (G*power 3.1, University of Duesseldorf, Germany) (Faulkner et al. 2013a, b). Participants were informed of the benefits and risks of the study prior to giving their written informed consent to participate in the study. They completed a general health-screen questionnaire and were all non-smokers and free from injury for 6 months before their first visit. Prior to any experimental activity, the study was approved by the institutional ethics committee and all procedures were conducted according to all aspects of the Declaration of Helsinki, apart from registration in a database.

### Study overview

This study used a within-participant, randomised experimental design. Each participant was required to visit an environmental chamber (TIS Services, Alton, Hampshire, UK) on five separate occasions, including one familiarisation session, with each session ~ 7 days apart. Trials were performed at the same time of day (± 1 h) to minimise circadian effects and prior to the experimental visits, participants were familiarised with the experimental protocol along with demographic data being recorded. During the four experimental visits, upon arrival, participants completed a 15-min standardised pre-match warm-up on an indoor sprint track (~ 18 °C). Following this, participants entered the environmental chamber, set at the two environmental conditions: (1) a thermoneutral environment (18 °C) or (2) a cold environment (2 °C). In each environmental condition, participants wore a tracksuit top and either a pair of standardised tracksuit bottoms (CON) or, a pair of externally heated trousers (HUUB Design, Derby, UK; HEAT) over standard soccer attire. Following the 45-min passive substitute simulation, players returned to the indoor sprint track to complete a second-half re-warm-up and then returned to the environmental chamber for the simulated 2nd half (45 min). Therefore, the four conditions were HEAT in 18 °C (HEAT18), CON in 18 °C (CON18), HEAT in 2 °C (HEAT02) and CON in 2 °C (CON02).

### Procedure

Upon arrival, participants were dressed in typical match day apparel and laid on a plinth outside the environmental chamber. During this time, guided by ultrasound, participants were marked using a sterile pen, at two locations on the right thigh, where temperature probes would be inserted. Subsequently, a heart rate (HR) monitor was fitted (Polar FT1; Polar Electro155 Oy, Kempele, Finland). Participants then completed the 15‐min soccer-specific warm‐up. The 15‐min protocol included a combination of bodyweight exercises, as well as ballistic and plyometric movements, performed over a 20 m circuit utilising a Raise, Activate, Mobilise, Potentiate (RAMP) method (Jeffreys 2007). The participants were instructed to perform two sets of jogging (40 m) and skipping (40 m), followed by one set (20 m) of each dynamic stretch exercise (knee raises; heel flicks; lateral side lunges; front lunges; high kicks; tuck jumps; and reactive sprints). Following the completion of the dynamic stretches, participants had to complete two sets each of straight sprinting, tuck jump into sprints and opponent reaction sprints (Fashioni et al. 2020).

Following the active warm‐up, participants entered the environmental chamber and laid supine on a plinth before a temperature probe (MAC‐07170‐A, Ellab, Manchester, UK) was inserted at the first insertion mark (further details below) into the VL at a 2 cm depth. An aural thermistor (Grant Instruments, Cambridge, UK) was placed ~ 3 cm in the ear canal and was used as the measure of T_core_; both muscle and aural probes were connected to a data logger (Squirrel SQ2020 Data Logger, Dorset, UK) that sampled data in 10-s epochs. Participants were then seated for 45 min, simulating a substitute for the 1st half of a soccer match.

After the 45 min of passive rest, the tracksuit, muscle temperature probe and aural thermistor were removed and the participants returned to the indoor track to perform a 15-min re-warm‐up, simulating the half‐time period. Participants performed light ball work for the initial 5 min; in the following 7 min participants performed the soccer‐specific aerobic field test agility course, involving a repeated 20‐m soccer‐specific runs based on targeting to maintain 70% HR_Max_ (Edholm et al. 2014; Lovell et al. 2013; Mohr et al. 2004), allowing 3 min to travel to and from the indoor running track. Following this, participants again entered the environmental chamber for an additional 45 min to replicate the role of a substitute for the 2nd half of a match.

**Fig. 1:**
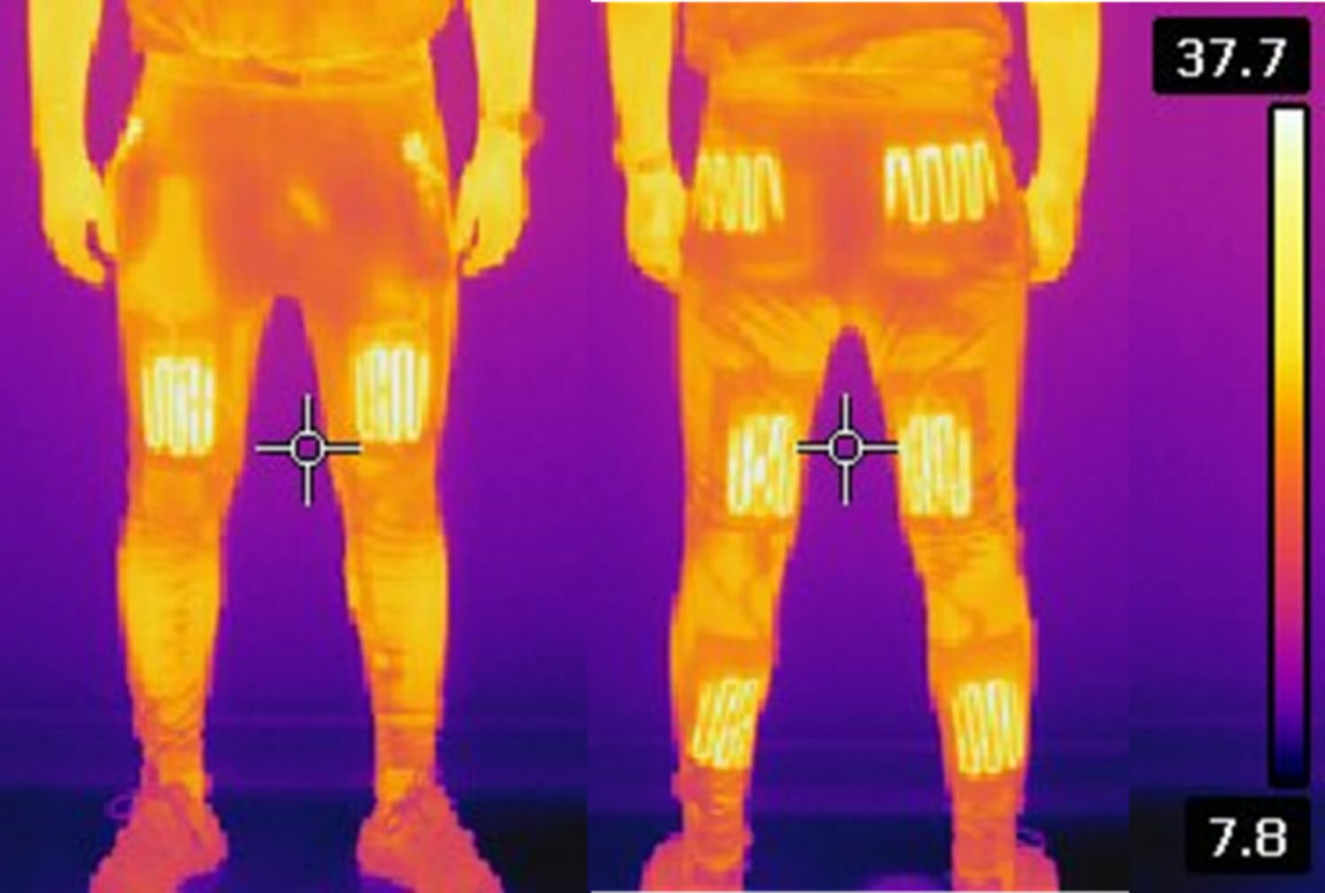
Thermogram of the Huub Design Heated Trousers showing an anterior view on the left and posterior on the right

Following the end of the 2nd half protocol, the temperature probe and aural thermometer were removed. Throughout the passive rest phases, participants wore either HEAT or CON. Both trousers had similar insulation when unheated. The heated trousers (HUUB Design, Derby, UK) were chosen because of the optimal coverage of the quadriceps, hamstrings, calves, and glutes with the heating elements (see Fig. 1) (Faulkner et al. 2013a, b). The trousers stretch panels allowed for optimal heat transfer, as the material is maintained close to the body, thus decreasing convection whilst allowing movement. The maximum temperature of the heating elements was 45 °C. Based on similar heated trousers, when the heating elements were inactive (CON), the insulation value was approximately 0.559 m^2^·K·W^–1^ (3.6 clo) for the legs and hips in isolation that were covered by the garment. When the heating elements were initiated (HEAT), insulation of the legs and hips increased to give a local insulation value of approximately 0.842 m^2^·K·W^–1^ (5.4 clo) (Faulkner et al. 2013a, b). During the periods of passive rest, every 5 min T_muscle_ and *T*_core_ as well as thermal comfort (TC) and thermal sensation (TS) using visual analogue scales was recorded (Gagge et al. 1967).

### Muscle temperature measurement

VL T_muscle_, at a depth of 2 cm, was recorded during the trouser intervention using calibrated, sterile, flexible intramuscular finewire thermocouple probes (MAC-07170-A, Ellab, Copenhagen, Denmark). An ultrasound (Logiq E BT12, General Electric, Duluth, MN) and 8 cm linear transducer (12 L-RS, General Electric, Duluth, MN) was used to accurately measure subcutaneous fat and the correct depth of the probe insertion. The ultrasound was also used to identify the exact site of insertion, the centre of the VL. To ensure consistent and accurate needle insertion placement, a measurement was taken midway between the greater trochanter and the lateral epicondyle of the femur to find the midpoint of the VL. Then, the VL was marked in the centre of the lateral and medial border using a sterile pen; additional markings were placed 1-cm inferior and superior to this point, for each insertion point.

For muscle probe insertion, first, pre-injection swabs were used to clean the area of insertion. Using ultrasound guidance, a sterile 18G hypodermic safety needle catheter was inserted 2 cm into the VL. Once a depth of 2 cm was reached, a thermocouple probe with a diameter of 0.7 mm was inserted through the needle catheter. Once the thermocouple probe was placed through the needle catheter, at the depth of 2 cm, the hypodermic needle catheter was removed, whilst the temperature probe remained embedded in the muscle at the required depth. The remainder of thermocouple probe was then secured by medical tape on the skin, to secure the probe in place. The thermocouples were then connected to a data logger (Squirrel SQ2020 Data Logger, Dorset, UK) using crocodile clips for continuous recording. Temperature probes remained inserted throughout the duration of the passive protocol and were removed prior to the second-half warm-up and reinserted immediately post second-half warm-up. Throughout the muscle temperature measurements, due to the participants experiencing limited discomfort, it was deemed unnecessary for any form of anaesthetic to be used. Insertion of the probe took ~ 150 s following cessation of the pre-match and 2nd half warm-ups.

### Core temperature and perceptual measures

Prior to the passive protocol, an aural thermistor (Grant Instruments, Cambridge, UK) was inserted into the participant’s right auditory canal (Benzinger and Taylor 1963) to measure *T*_core_. This was recorded throughout the passive rest phases of the protocol, the aural thermistor was securely taped into position and insulated with cotton wool, before a headband was fitted to maintain placement (Barwood et al. 2014). The aural thermistor was also connected to the data logger (Squirrel SQ2020 Data Logger, Dorset, UK).

Perceptual measures including thermal comfort (TC) and sensation (TS) were taken throughout passive rest periods using visual analogue scales (Gagge et al. 1967). The number range for both scales are consistent, but anchors varied (TC, − 3 very uncomfortable, − 2 uncomfortable, − 1 just uncomfortable, 0 neutral, 1 just comfortable, 2 comfortable, 3 very comfortable: TS, − 3 cold, − 2 slightly cold, − 1 cool, 0 neutral, 1 warm, 2 slightly hot, 3 hot). Participants were asked to ensure the trousers felt ‘comfortable (≤ 2)’ and ‘hot (≤ 3)’, if the participant felt ‘uncomfortable (≥ − 2)’ the heat stimulus was reduced (3 participants required the heat to be reduced after 10 ± 5 min). If participants felt ‘cold (≥ 3)’ extra clothes including hat and gloves were provided (4 participants required extra clothes at 22.5 ± 9.6 min), which was replicated for each half of the trial and for each individual environmental condition.

### Data analysis

All statistical tests were processed using IBM SPSS Statistics 22 (SPSS Inc., Chicago, IL). Parameters measured throughout the passive rest period (TS, TC, T_core_, T_muscle_) were analysed (two-tailed) following the 1st and 2nd halves using a two-way (trial [2] × time [4]) Analysis of Variance (ANOVA) with multiple comparisons corrected using the Tukey method when significant main or interaction effects were observed. The accepted level of significance was *P* < 0.05. Data are presented as mean ± SD.

## Results

### 2 °C condition

#### Muscle temperature

**Fig. 2:**
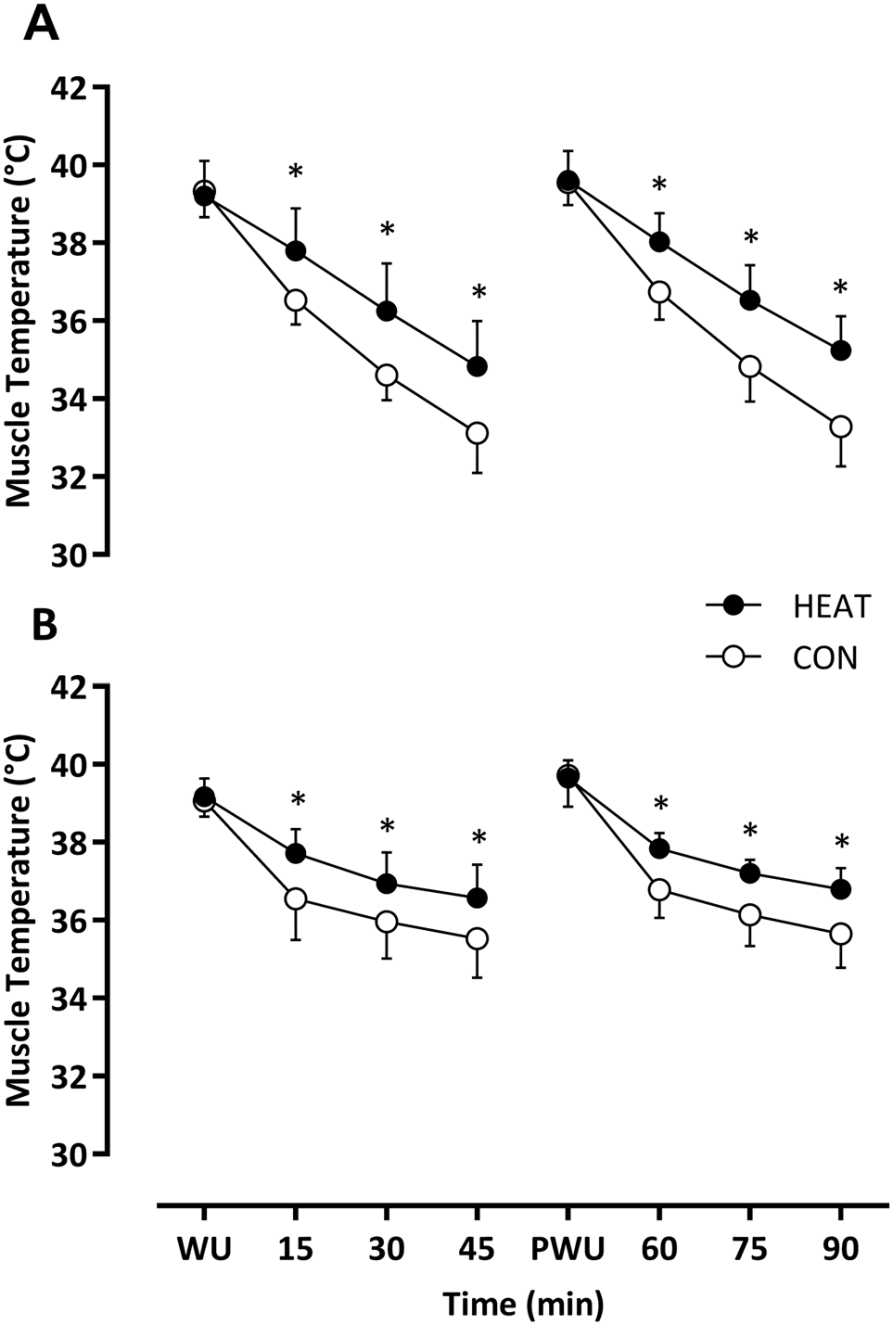
Measurement of muscle temperature in an environmental condition of 2 °C (**A**) and 18 °C (**B**) throughout the 90 min of passive recovery. Data presented as mean ± SD. *P < 0.05 vs. the same timepoint in CON

Baseline T_muscle_ was similar (HEAT: 39.1 ± 0.5 vs. CON: 39.3 ± 0.7 °C; *P* = 0.79). Throughout the simulated 1st half, T_muscle_ reduced over time (*F*_3,21_ = 321, *P* < 0.001) and the decline was attenuated in HEAT vs. CON (Δ − 4.39 ± 0.81 vs. − 6.21 ± 1.32 °C; *F*_1,7_ = 21.2, *P* = 0.002). Furthermore, the rate of change in T_muscle_ differed over time between HEAT and CON (*F*_3,21_ = 2.9, *P* < 0.001), with post hoc analyses confirming that, T_muscle_ in HEAT was higher in the 1st half at 15 (Δ 0.96 °C; *P* = 0.010), 30 (Δ 1.10 °C;* P* < 0.001), and 45 min (1.16 °C; *P* < 0.001) vs. CON (Fig. 2A). After the half-time warm-up, T_muscle_ was comparable between conditions and similar to baseline (HEAT: 39.6 ± 0.8 vs. CON: 39.6 ± 0.6 °C; *P* = 0.862). During the 2nd half, T_muscle_ reduced (*F*_3,21_ = 429, *P* < 0.001) and the decline was attenuated in HEAT vs. CON (Δ − 4.36 ± 1.03 vs. − 6.26 ± 1.04 °C; *F*_1,7_ = 23.3, *P* = 0.002). Furthermore, the rate of change in T_muscle_ over time differed between conditions (*F*_3,21_ = 13.63, *P* < 0.001), with post hoc analyses confirming higher T_muscle_ in HEAT vs. CON after 60 (Δ 1.25 °C; *P* < 0.001), 75 (Δ 1.26 °C; *P* < 0.001), and 90 min (Δ 1.51 °C; *P* < 0.001) vs. CON (Fig. 2A).

#### Core temperature

**Fig. 3:**
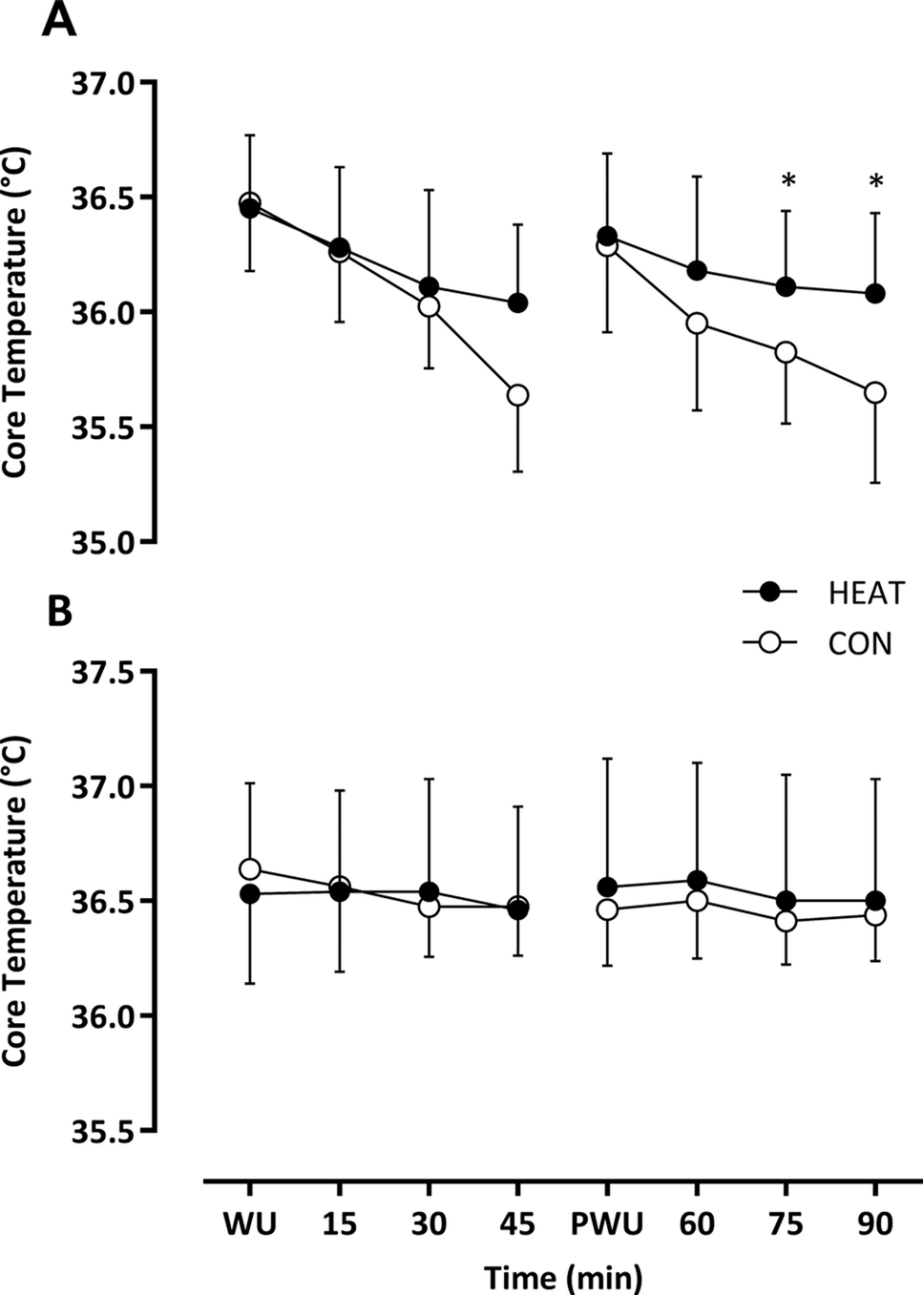
Measurement of core temperature in an environmental condition of 2 °C (**A**) and 18 °C (**B**) throughout the 90 min of passive recovery. Data presented as mean ± SD. *P < 0.05 vs. the same timepoint in CON

Baseline T_core_ was similar in each condition (HEAT: 36.45 ± 0.32 vs. CON 36.48 ± 0.3 °C; *P* = 0.79). Throughout the 1st half, T_core_ reduced over time (*F*_3,21_ = 20.48, *P* < 0.001) but the decline was attenuated in HEAT vs. CON (Δ − 0.41 ± 0.25 vs. Δ − 0.84 ± 0.41 °C; *F*_1,7_ = 6.63; *P* = 0.037). Furthermore, the rate of change in T_core_ differed over time between HEAT vs. CON (*F*_3,21_ = 3.15, *P* = 0.047). After the 2nd half warm-up T_core_ was comparable (HEAT: 36.33 ± 0.36 °C vs. CON: 36.29 ± 0.38 °C; *P* = 0.67) and reduced throughout the 2nd half (*F*_3,21_ = 16.59, *P* < 0.001), a reduction that was again attenuated in HEAT vs. CON (Δ − 0.25 ± 0.33 vs. Δ − 0.64 ± 0.34 °C; *F*_1,7_ = 7.67; *P* = 0.028). Furthermore, the rate of change in T_core_ over time was attenuated in HEAT vs. CON (*F*_3.21_ = 6.14, *P* = 0.025), with post hoc analyses confirming that T_core_ was higher in HEAT after 75 (Δ 0.29 °C; *P* = 0.019) and 90 min (Δ 0.43 °C; *P* = 0.01) (Fig. 3A).

#### Thermal comfort and thermal sensation

Perceptual measures were similar at baseline in each condition but throughout the simulated 1st half, TC (*F*_3,21_ = 20.48, *P* < 0.001) and TS (*F*_3,21_ = 42.3, *P* < 0.001) reduced over time. Following the half-time warm-up, TC and TS were comparable. TC (*F*_3,21_ = 16.59, *P* < 0.001) and TS (F_3,21_ = 36.4, *P* < 0.001) both reduced throughout the 2nd half and post hoc analyses confirmed that participants felt “more comfortable” and “warmer” after 75 (TC: *P* = 0.019; TS: *P* = 0.031) and 90 min (TC: *P* = 0.01; TS: *P* = 0.002) in HEAT vs. CON (Table 1).

**Table 1:** Thermal comfort and thermal sensation throughout the passive rest intervention periods

Condition	PWU	15	30	45	PWU	60	75	90
Thermal Comfort (TC)								
2 °C CON	2.0 ± 0.8	0.4 ± 1.7	– 0.3 ± 1.5	– 1.0 ± 1.1	1.6 ± 0.7	– 0.3 ± 1.6	– 0.9 ± 1.6	– 1.3 ± 1.5
2 °C HEAT	2.3 ± 0.7	1.5 ± 1.3	0.8 ± 1.5	0.6 ± 1.5	1.8 ± 0.9	1.5 ± 1.1	1.3 ± 0.9*	1.0 ± 0.9**
18 °C CON	1.6 ± 0.7	1.6 ± 0.7	1.5 ± 0.8	1.4 ± 0.9	1.8 ± 0.5	1.6 ± 0.5	1.6 ± 0.7	1.6 ± 0.7
18 °C HEAT	1.9 ± 0.8	2.1 ± 0.6	1.8 ± 0.5	1.8 ± 0.5	1.9 ± 1.0	2.0 ± 0.8	1.9 ± 0.6	1.9 ± 0.6
Thermal Sensation (TS)								
2 °C CON	1.5 ± 0.5	– 0.8 ± 1.3	– 1.5 ± 1.2	– 1.9 ± 1.0	1.4 ± 0.7	– 0.8 ± 1.0	– 1.6 ± 1.2	– 2.3 ± 1.0
2 °C HEAT	1.6 ± 0.7	0.6 ± 1.4	– 0.1 ± 1	– 0.4 ± 1.3	1.4 ± 0.7	0.8 ± 1.3	0.1 ± 1*	0.0 ± 1.2**
18 °C CON	1.5 ± 0.8	0.5 ± 0.8	0.3 ± 0.5	0.3 ± 0.5	1.6 ± 0.9	0.8 ± 0.7	0.6 ± 0.7	0.6 ± 0.7
18 °C HEAT	1.6 ± 0.7	1.4 ± 0.5	1.4 ± 0.5**	1.4 ± 0.5**	1.6 ± 0.7	1.5 ± 0.8	1.5 ± 0.8	1.5 ± 0.8

Visual analogue scale anchors: TC, – 3 very uncomfortable, – 2 uncomfortable, – 1 just uncomfortable, 0 neutral, 1 just comfortable, 2 comfortable, 3 very comfortable; TS, – 3 cold, – 2 slightly cold, – 1 cool, 0 neutral, 1 warm, 2 slightly hot, 3 hot

*PWU* Post warm-up

*P < 0.05 **P < 0.01 of HEAT vs. CON in the specific environmental condition

### 18 °C condition

#### Muscle temperature

Baseline temperature was similar in each condition (HEAT: 39.2 ± 0.5 vs. CON: 39.1 ± 0.4 °C; *P* = 0.575), however, reduced over time throughout the 1st half (*F*_3,21_ = 203, *P* < 0.001) and the decline was attenuated in HEAT vs. CON (Δ − 2.48 ± 0.71 vs. Δ − 3.54 ± 0.88 °C; *F*_1,7_ = 18.81, *P* = 0.003). Furthermore, the rate of change in T_muscle_ differed over time in HEAT vs. CON (*F*_3,21_ = 6.42, *P* = 0.003) with post hoc analyses confirming that, T_muscle_ in HEAT was higher in the 1st half at 15 (Δ 1.16 °C; *P* = 0.015), 30 (Δ 0.99 °C; *P* = 0.042), and 45 min (1.06 °C; *P* = 0.035) vs. CON. After the half-time warm-up, T_muscle_ was comparable between conditions and similar to baseline (HEAT: 39.6 ± 0.8 vs. CON: 39.6 ± 0.6 °C; *P* = 0.862). T_muscle_ reduced throughout the 2nd half (*F*_3,21_ = 376, *P* < 0.001); however, the decline was attenuated in HEAT vs. CON (Δ − 2.85 ± 0.57 vs. Δ − 4.06 ± 1 °C; *F*_1,7_ = 9.44, *P* = 0.018). Furthermore, the rate of change in muscle temperature over time varied (*F*_3,21_ = 5.89, *P* = 0.004) with post hoc analyses confirming higher T_muscle_ in HEAT vs. CON after 60 (Δ 1.05 °C; *P* = 0.035), 75 (Δ 1.04 °C; *P* = 0.032) and 90 min (Δ 1.05 °C; *P* = 0.004) (Fig. 2B).

#### Core temperature

Temperature at baseline was similar in each condition (HEAT: 36.53 ± 0.39 vs. CON: 36.53 ± 0.37 °C; *P* = 0.47) and throughout the 1st half, T_core_ was comparable between HEAT and CON (*F*_3,21_ = 1.16, *P* = 0.341). A small decline in T_core_ was observed, which was similar between HEAT and CON (Δ − 0.06 ± 0.41 vs. Δ − 0.16. ± 0.39 °C; *F*_1,7_ = 0.265;* P* = 0.623; interaction effect, *F*_3,21_ = 0.329, *P* = 0.651). Following the half time warm-up, T_core_ was comparable between conditions and similar to baseline (HEAT: 36.56 ± 0.56 vs. CON: 36.46 ± 0.24 °C; *P* = 0.62). Furthermore, T_core_ was similar throughout the 2nd in HEAT vs. CON (*F*_3,21_ = 0.421, *P* = 0.74, Δ − 0.06 ± 0.59 vs. Δ 0 ± 0.27 °C; interaction effect, *F*_1,7_ = 0.45; *P* = 0.839) (Fig. 3B).

### Thermal comfort and thermal sensation

TC and TS were similar at baseline in each condition. Throughout the 1st half, TC (*F*_3,21_ = 0.866, *P* = 0.407) was comparable between conditions; however, TS reduced over time (*F*_3,21_ = 16.9, *P* < 0.001). Following the half time warm-up, TC and TS were similar. TC was similar between HEAT and CON (*F*_3,21_ = 0.104, *P* = 0.957) but TS (*F*_3,21_ = 5.05, *P* = 0.009) reduced throughout the 2nd half and was again attenuated by the passive heat intervention (Δ − 0.13 ± 0.64 vs. Δ − 1 ± 0.93; *F*_1,7_ = 7.61; *P* = 0.028). Post hoc analyses showed that in the 2nd half, participants felt “warmer” after 30 (*P* = 0.008) and 45 min (*P* = 0.008) (Table 1).

## Discussion

The aim of this study was to investigate the use of a passive heat intervention on T_muscle_ and T_core_ throughout a simulated soccer match for unused substitutes, in two (2 and 18 °C) environmental conditions commonly experienced throughout the UK soccer season. The main finding of the present study was that the decline in T_muscle_ and T_core_ experienced by substitutes in the period of inactivity, can be attenuated using heated trousers. Specifically, the use of heated garments led to a reduced decline of muscle temperature by ~ 1.1 °C at the end of the 1st half and ~ 1.2 °C following the 2nd half in 18 °C. The attenuation was greater when passive heat was used during the cold environment (2 °C), with ~ 1.8 °C difference following the 1st half and ~ 1.9 °C after the 2nd half. Interestingly, the passive heat intervention also impacted systemic thermoregulation with an effect on core temperature in the cold, but this was not evident in a thermoneutral environment, despite reductions in muscle temperature during the period of inactivity. Thus, the present study suggests that the use of a heated trouser intervention can partially mitigate the reduction in muscle temperature that is associated with periods of inactivity. Our data confirm how such an intervention might be advantageous for substitutes entering a match scenario as they would experience a reduced drop in muscle temperature following pre-match and half-time warm-ups especially in the cold.

Our findings support the suggestion of a review conducted by Silva et al (2018), that the application of heated garments following an active warm-up is imperative to maintain muscle temperature (Silva et al. 2018). Furthermore, Faulkner and colleagues found that following an active warm-up, the use of electrically heated trousers during a 30-min passive transition period in an ambient temperature, can reduce the decline in T_muscle_ (HEAT: − 1.2 ± 0.2 vs. CON: − 1.5 °C; *P* < 0.05) (Faulkner et al. 2013a, b). However, aligned to the current study, although the decline in muscle temperature was reduced, a decline in T_muscle_ was still apparent. This may be attributed to some limiting factors, including the cooling effect of blood flow to the VL, this could be from arterial blood from the core, venous blood from the lower leg, or both (Raccuglia et al. 2016). Another influence which might reduce muscle temperature is the microclimate between the heated trousers and the skin, which is exaggerated in colder environments [19]. Bespoke high-density water-perfused trousers (WPT; Med-Eng System Inc., Pembroke, Canada) decreased the microclimate between the trousers and the skin and exhibited a substantial reduction in the muscle temperature drop compared to previous studies which used trousers with electrically powered heating elements (Faulkner et al. 2013a, b; Raccuglia et al. 2016). However, given the water-perfused pants were connected to a heating system, consisting of a temperature-controlled water bath and powered water pump, in its current state, it may not be practical for field-based sports, specifically to enhance soccer performance (Raccuglia et al. 2016). Therefore, electrically heated garments appear to be a more suitable method for passively heating the muscle before field-based exercises, hence the application of the HUUB heated trousers (HUUB Design, Derby, UK) for this study. Moreover, our data confirms just how sensitive periods of inactivity are for muscle temperature.

Limited studies have determined the physiological outcomes of warming-up passively for long duration performances. This might be because a limiting factor to long-duration performances is excessive bodily heat (Kozlowski et al. 1985; Romer et al. 2001). Consequently, a rise in T_core_ before exercise might be detrimental to long duration performance due to impaired thermoregulatory mechanisms (Fortney et al. 1984) and/or a decrease in heat storage capacity (Nadel 1987). Thus, in terms of the decline in T_muscle_ and T_core_, environmental conditions are a crucial factor to be considered. This present study observed that when heated pants are utilised following an active warm-up in a cold environment, T_core_ and T_muscle_ would be significantly lower in comparison with when the same protocol was applied in ambient conditions (18 °C). Therefore, in a colder environment, the time to reach a critical core temperature would likely be delayed, and the opportunity for performance improvement might be exaggerated (Cowper et al. 2021).

As a T_core_ maintenance strategy, the present study demonstrated that in a cold environment, HEAT attenuated the reduction of T_core_ after 30 min of the 2nd half compared to CON. This is aligned with Cowper et al. (2021) who reported a significant improvement of T_core_ in HEAT compared to CON by 1.47 °C following a passive rest period of 25 min (Cowper et al. 2021). However, the present study demonstrates that in a thermoneutral environment, T_core_ does not change throughout the passive rest period following the use of heated trousers. This was also apparent in several studies which have utilised heated garments during a passive recovery period (Faulkner et al. 2013a, b; Wilkins and Havenith 2017). Although, this study presents in a thermoneutral environment, T_core_ is maintained throughout, whilst T_muscle_ is attenuated with HEAT compared with CON. Therefore, this give scope for HEAT to be considered at any temperature ≤ 18 °C.

Utilising heated trousers appears to not only enhance T_muscle_ and T_core_, but this present study has shown the intervention can also improve TC levels within soccer substitutes in a cold condition. Improvements in TC pre-exercise has been found to subsequently improve sporting performance (Schlader et al. 2011). In line with the perceptual data from this present study, ratings of TC and TS improved when using the heated trousers (Table 1), suggesting that participants felt more comfortable in this trial. Indeed, being warm causes widespread changes in the central nervous system (Lowry et al. 2009) and increases perceptions of readiness to perform (Wilkins and Havenith 2017). Thus, further research is required to access the effect on performance, this appears to be a positive indication that heated garments will not hinder performance and may improve performance parameters with the increased thermal comfort in colder environments. Overall, the data presented in this study may have important implications for a range of team sports, where substitutes remain inactive for extended periods of time.

Although performance was not measured in this study, it is well-established that muscle temperature and muscle function are related (Bergh and Ekblom 1979; Davies et al. 1982; Sargeant 1987). The attenuation in muscle temperature when using the passive heat intervention may lead to an improved performance via improved myosin adenosine triphosphatase activity. Thereby increasing the rate of ATP turnover and calcium sequestration by the sarcoplasmic reticulum, which in turn increases overall power output in the muscles in comparison with cooler states (Gray et al. 2006; Racinais and Oksa 2010; Stein et al. 1982). Given that muscular power is key for soccer performance (Mohr et al. 2004), it is vital that T_muscle_ is maintained. This highlights the importance that this present study demonstrated that in a 2 °C environment, attenuation of muscle temperature was evident as early as 15 min after an active warm-up following the heated intervention. It is well-established that one of the primary functions of a warm-up prior to power- or sprint-based activities is to increase muscle temperature (Asmussen and Bøje 1945; Faulkner et al. 2013a, b; Sargeant 1987). Mohr et al. (2004) found that in soccer players, an increase in muscle temperature following the half-time period, significantly improved sprint performance (Mohr et al. 2004). Such findings highlight the importance of the data in the present study. The use of heated garments, whilst substitutes are inactive, enables the players to utilise their physical capacities to a greater extent at the onset of match-play (Mohr et al. 2004).

### Limitations

A possible limitation to this study is the accuracy and repeatability of the 2 cm T_muscle_ measurement depth of the VL. However, the use of ultrasound prior to the intramuscular insertion allowed consideration of the additional subcutaneous fat depth needed to insert the probe 2 cm into the VL. In addition, the time taken to return to the environmental chamber and insert the muscle temperature probe, took ~ 150 s. Therefore, it is a possibility that the T_muscle_ might have been slightly underestimated, in the early stages of the passive recovery protocol in all conditions. However, due to the nature of the flexible probe, it would not have been viable to perform the probe insertion prior to the warm-ups and for it to remain in place throughout; it would have increased the risk of the probe breaking due to the intense nature of the warm-up protocols. A further limitation was the inability to perform the active warm-up and the re-warm-up in the environmental chamber; therefore, the warm-ups had to be performed at room temperature on an indoor sprint track (~ 18 °C).

### Practical implications

In team sports, even at the highest level of competition, during match-play substitutes are often sitting on the bench or performing low intensity stretches on the side-line. Considering the results of the present study, this leaves substitutes with significantly lower muscle temperatures which is further exacerbated in colder environments. When using a passive heat intervention, not only might the players perform better physically, but they might enter a match play scenario with a greater degree of ‘‘readiness’’. This may increase the probability that the substitute may perform better at the start of match-entry, which could affect the play throughout the rest of the game. In addition, using passive heat on the substitutes bench may act as an alternative to repeated high-intensity sprints prior to entering match-play, which has the potential to elicit fatigue (Goodall et al. 2015; Mohr et al. 2004).

## Conclusion

This study demonstrates that after active pre-match and half-time warm-ups, the application of heated garments in both ambient and cold environments led to a reduced drop in muscle temperature and thereby provides a scope for a potential improvement in soccer performance. The findings reported here, might be applicable to other sports, where substitutes experience lengthy passive rest periods post warm-up and in particular, events which are frequently performed in the cold.

## Data Availability

Data are not publicly available.

## Abbreviations

ANOVA: Analysis of Variance
HR: Heart Rate
RAMP: Raise, Activate, Mobilise, Potentiate
TC: Thermal Comfort
TS: Thermal Sensation
T_Core_: Core Temperature
T_Muscle_: Muscle Temperature
VL: Vastus lateralis
WU: Warm-up
